# An Observational Study of Vaping Knowledge and Perceptions in a Sample of U.S. Adults

**DOI:** 10.7759/cureus.8800

**Published:** 2020-06-24

**Authors:** Alexandra Bellisario, Karissa Bourbeau, Danielle A Crespo, Nicole DeLuzio, Alexandra Ferro, Alexandra Sanchez, Tracy Jackson, Gail Kunath-Tiburzi, Anthony V D'Antoni

**Affiliations:** 1 Physician Assistant Program, Wagner College, Staten Island, USA; 2 Radiology, Weill Cornell Medicine, New York, USA

**Keywords:** addiction, electronic cigarettes, lung injury, pulmonary, vaping, e-cigarette and vaping product use associated lung injury (evali)

## Abstract

Background

Vaping is the use of e-cigarettes that contain inhalants such as nicotine, tetrahydrocannabinol, and cannabidiol. Vaping is associated with e-cigarette or vaping product use associated lung injury (EVALI) and is a recognized public health crisis. Despite rising numbers of hospitalizations due to EVALI, public knowledge and perceptions of the dangers of vaping require further investigation.

Objectives

This exploratory study assessed knowledge and perceptions of vaping in U.S. adults.

Methods

This study was approved by an ethical board, and informed consent was obtained from all participants. A cohort of U.S. adults was recruited by shared links on social media. Participants completed an anonymous online survey that contained vaping knowledge and perceptions items. An a priori power analysis was conducted at 95% power and alpha = 0.05. Statistics were calculated using IBM SPSS Statistics Version 26 (IBM Corp., Armonk, NY, USA).

Results

A sample of 413 (N = 413) U.S. adults participated in the survey. The majority of participants (79.18%) were females, and 65.62% were between 18 and 24 years of age. Over half (62.71%) of participants were never asked about vaping use by a clinician at any visit, and 56.51% agreed that vaping can reduce stress. Of all participants, 70.91% agreed that drinking alcohol makes someone more inclined to vape. Significant positive Spearman’s rho correlations were found between vaping and the use of cannabis, cocaine, ecstasy, hallucinogens, and inhalants (p < 0.05).

Conclusions

We found a significant correlation between vaping and drug use. We also found that if the dangers of vaping are discussed by their health care providers, participants are more inclined to quit vaping. Unfortunately, many physicians report that they avoid discussing vaping with their patients due to lack of vaping knowledge. Our results illuminate the communication gap between patients and physicians. All clinicians need to counsel patients on the dangers of vaping, which might help prevent EVALI and related conditions.

## Introduction

Vaping is inhaling smoke from electronic cigarettes (e-cigarettes) that may contain nicotine, tetrahydrocannabinol, and cannabidiol [1]. Vaping is now recognized as a global public health crisis [2]. Vaping is associated with harmful conditions that include e-cigarette or vaping product use associated lung injury (EVALI) [1,3]. Despite the rising numbers of hospitalizations due to EVALI [1], public knowledge and perceptions of the dangers of vaping are still not clear as the incidence of vaping continues to rise in children and young adults [4].

Using a murine model, pulmonary responses to e-cigarettes were assessed and it was found that mice exposed to e-cigarettes over only a two-week period produced significant increases in pulmonary oxidative stress and moderate macrophage-mediated inflammation compared to placebo (p < 0.05) [5]. These authors concluded that e-cigarette vapor is a source of free radicals in which exposure can cause airway inflammation, oxidative stress, and suppresses bacterial clearance by alveolar macrophages [5]. Other researchers analyzed the tumorigenicity of e-cigarette smoke on lung and bladder tissue in mice [6]. They found that 22.5% of mice exposed to e-cigarette smoke developed lung tumors (adenocarcinomas) and 57.5% developed urothelial hyperplasia in their urinary bladders [6]. These data from basic science studies correlate with recent clinical findings. In the final analysis of their originally published case series, researchers stated that 98 patients (N = 98) in Wisconsin and Illinois were reported to their respective public health departments due to EVALI [7]. The patients had bilateral infiltrates on chest imaging as a result of vaping. A total of 95% of the patients were hospitalized, 26% underwent intubation and mechanical ventilation, and two deaths were reported [7]. A total of 89% of the patients reported having used tetrahydrocannabinol products in e-cigarette devices, although a wide variety of products and devices was reported [7]. Using a cross-sectional survey of 8,087 participants (N = 8,087), Wills et al. [8] found a significant association of e-cigarette use with chronic pulmonary disorder (p < 0.01). Others recently analyzed bronchoalveolar lavage fluid from a convenient sample of 51 patients (N = 51) with EVALI to quantify the degree of toxicants and their chemical effects on lung tissue [3]. These researchers found that vitamin E acetate was associated with EVALI [3]. Clearly, vaping is not an innocuous activity, and there exists a continued need to ascertain the perceptions of people who vape. Such data can help drive evidence-based public health initiatives.

Vape products come in a variety of styles, and there are over 7,000 available flavors in the market [9-11]. A cross-sectional survey in a large cohort (N = 728) was carried out to examine the relationship between product characteristics and e-cigarette appeal [12]. Of participants that exclusively vaped, 68.9% reported that the option of different flavors was the most attractive characteristic of using vapes that influenced their decision to begin vaping [12]. These results suggest that people who have never vaped or smoked cigarettes may be vulnerable to e-cigarette flavor marketing strategies. Allen et al. [9] analyzed the contents of 51 types of flavored e-cigarettes and found that diacetyl was detected above the laboratory limit of detection in 39 of 51 flavors (up to 239 µg/e-cigarette), 2,3-pentanedione was detected in 23 of 51 flavors (up to 64 µg/e-cigarette), and acetoin was detected in 46 of 51 flavors (up to 529 µg/e-cigarette). These data have driven lawmakers in some countries to ban flavored e-cigarettes or restrict them from being sold to adolescents. Rates of e-cigarette use among high school students in the United States have strikingly increased from 1.5% in 2011 to 20.8% in 2018 [13], and these data have been corroborated in more recent studies [4]. In a qualitative study of young adults (N = 49), researchers [14] conducted focus groups and four main themes emerged: positive reinforcement, social benefits, negative effect reduction, and negative consequences. They found that many young adults were unsure of the negative consequences of vaping [14].

Vaping research is in its infancy, and there exist large gaps in the literature related to knowledge and perceptions of vaping among people of all ages. Therefore, the purpose of this exploratory study is to assess public knowledge and perceptions of vaping by surveying a cohort of U.S. adults. The results can help clinicians provide effective vaping cessation strategies for their patients and drive evidence-based public health interventions. Our three hypotheses are as follows:

1. There exists an association between knowledge of the chemicals found in vape pods and vape usage.

2. There exists a relationship between vaping and concomitant drug use.

3. There exists an inverse association between knowledge of the dangers of vaping and vape usage.

## Materials and methods

The study protocol was fully approved by the Wagner College, Staten Island, NY, USA. Informed consent was obtained by all participants prior to their participation in the study. The design was an exploratory, observational study with a sample size of 413 (N = 413) participants. An a priori power analysis using G-power version 3.1.9.6 revealed that the minimum sample size need to achieve significance was 317 participants at 95% power, effect size of 0.25, at an alpha level of 0.05 [15, 16]. Because we did not find a published survey instrument that specifically aligned with the purpose of our study, we developed our own. The complexity of measuring perceptions related to vaping has been discussed in the literature [17]. Researchers have suggested that survey instruments be developed as e-cigarette products evolve [17]. They summarized 371 e-cigarette perception items from seven research groups, and we adapted some of our items from their summary [17]. The survey instrument was first piloted on 235 (N = 235) participants so that the items and responses could be analyzed for inconsistencies and revised, if necessary. Inconsistencies included the use of ambiguous terms or the lack of operational definitions for others. The wording of any items that appeared vague were was changed by consensus agreement among the authors. None of the data from these piloted participants were included in the final total sample. The final survey instrument included demographic items, as well as, vaping knowledge and perception items (see Appendix). A Likert scale was used for the knowledge and perception items. These items were paired (both positively and negatively worded items) but spaced from each other on the survey instrument. The purpose of these items was to evaluate acquiescence bias, which we did not find. The variables measured by the survey instrument are shown in Table 1. The inclusion criteria were participants 18 years or older, participants who vape or do not vape, and completed surveys. The exclusion criteria were participants less than 18 years of age and incomplete surveys. All statistics were calculated using IBM SPSS Statistics Version 26 (IBM Corp., Armonk, NY, USA).

**Table 1:** Dependent and independent variables

Variable	Scale of measurement	Type of statistic
Dependent variable		
Vaping	Ordinal	Non-parametric
Flavor	Nominal	Non-parametric
Smoking history	Ordinal	Non-parametric
Reasons for vaping	Nominal	Non-parametric
Frequency of use	Ordinal	Non-parametric
Independent variable		
Age	Ordinal	Non-parametric
Gender	Nominal	Non-parametric
Race	Nominal	Non-parametric
Knowledge of vaping risk	Ordinal	Non-parametric
Education	Ordinal	Non-parametric

We distributed our electronic survey on a variety of social media websites using SurveyMonkey^Ⓡ^. These websites included Facebook as a primary source due to its popularity and number of users. Others included Reddit, YouTube, and Instagram.

## Results

**Figure 1:**
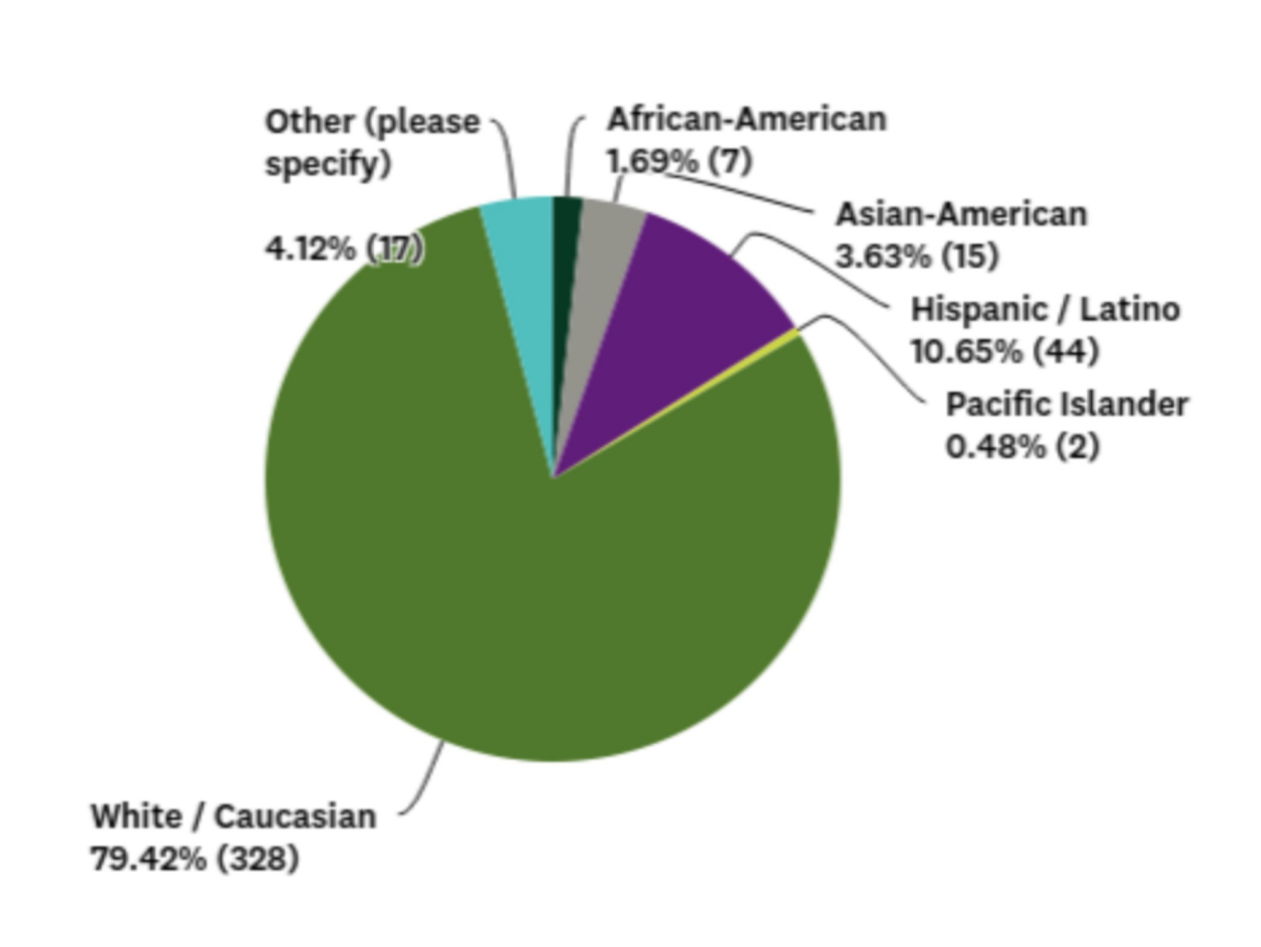
Pie chart demonstrating the ethnicity of the sample Data are shown as percentages (numbers).

**Figure 2:**
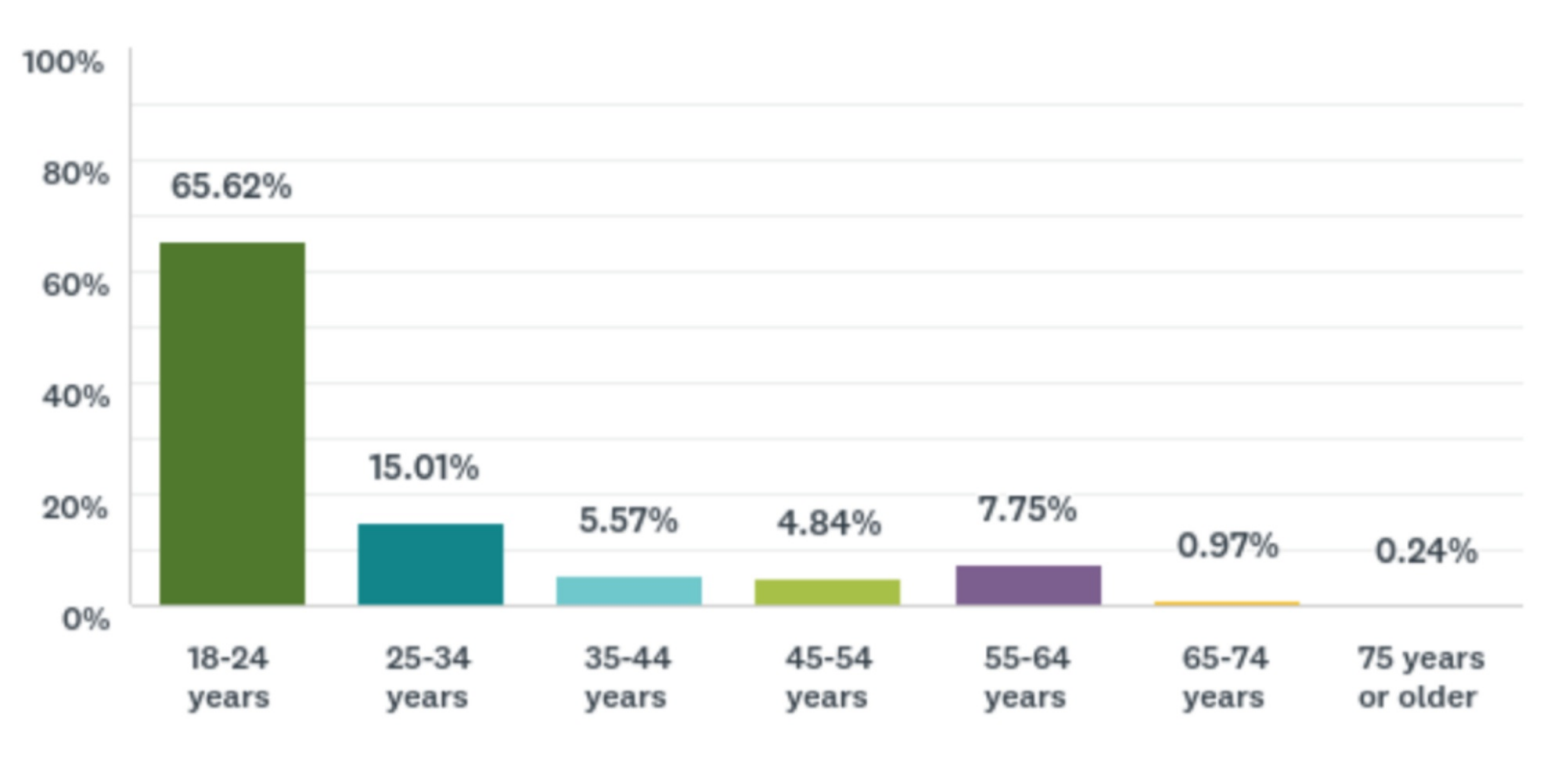
Bar chart demonstrating age range of the sample Data are shown as percentages.

A total sample of 413 (N = 413) U.S. adults participated. The internal consistency of our survey instrument was found to be moderately reliable (Cronbach’s alpha = 0.537). The gender and educational level of the sample are shown in Table 2. Most participants were females (79.18%) between the ages of 18 to 24 years (65.62%) and white/Caucasian (79.42%). Figures 1 and 2 depict these data. Table 3 includes the medical and psychiatric diagnoses of the sample.

**Table 2:** Gender and educational level of the sample (N = 413) ^a^Operational definition of gender variant/nonconforming: exhibiting behavioral, cultural, or psychological traits that do not correspond with the traits typically associated with one's sex; having a gender expression that does not conform to gender norms. GED, general educational development

Demographic variable	n (%)
Gender	
Male	82 (19.85%)
Female	327 (79.18%)
Transgender female	0 (0.00%)
Transgender male	1 (0.24%)
Gender variant/nonconforming^a^	2 (0.48%)
Not listed	1 (0.24%)
Prefer not to say	0 (0.00%)
Education level	
Some high school, no diploma	3 (0.73%)
High school graduate/diploma/GED	41 (9.93%)
Some college credits, no degree	140 (33.90%)
Trade school	0 (0.00%)
Associate’s degree	19 (4.60%)
Bachelor’s degree	124 (30.02%)
Master’s degree	67 (16.22%)
Doctoral degree (MD, DO, PhD, etc.)	19 (4.60%)

**Table 3:** Medical and psychiatric diagnoses of the sample (N = 413)

Demographic variable	n (%)
Medical diagnosis	
Asthma	88 (21.31%)
Chronic bronchitis	0 (0.00%)
Emphysema	0 (0.00%)
Lung cancer	0 (0.00%)
Reactive airway disease	3 (0.73%)
Recurrent pneumonia	2 (0.48%)
None of the above	320 (77.48%)
Psychiatric diagnosis	
Anorexia nervosa or bulimia	8 (1.94%)
Bipolar 1 or bipolar 2 disorder	2 (0.48%)
Generalized anxiety or panic disorder	85 (20.58%)
Major depressive disorder or seasonal depressive disorder	34 (8.23%)
Schizophrenia, schizophreniform, schizoaffective	0 (0.00%)
Substance use disorder	3 (0.73%)
Never been diagnosed	281 (68.04%)

**Figure 3:**
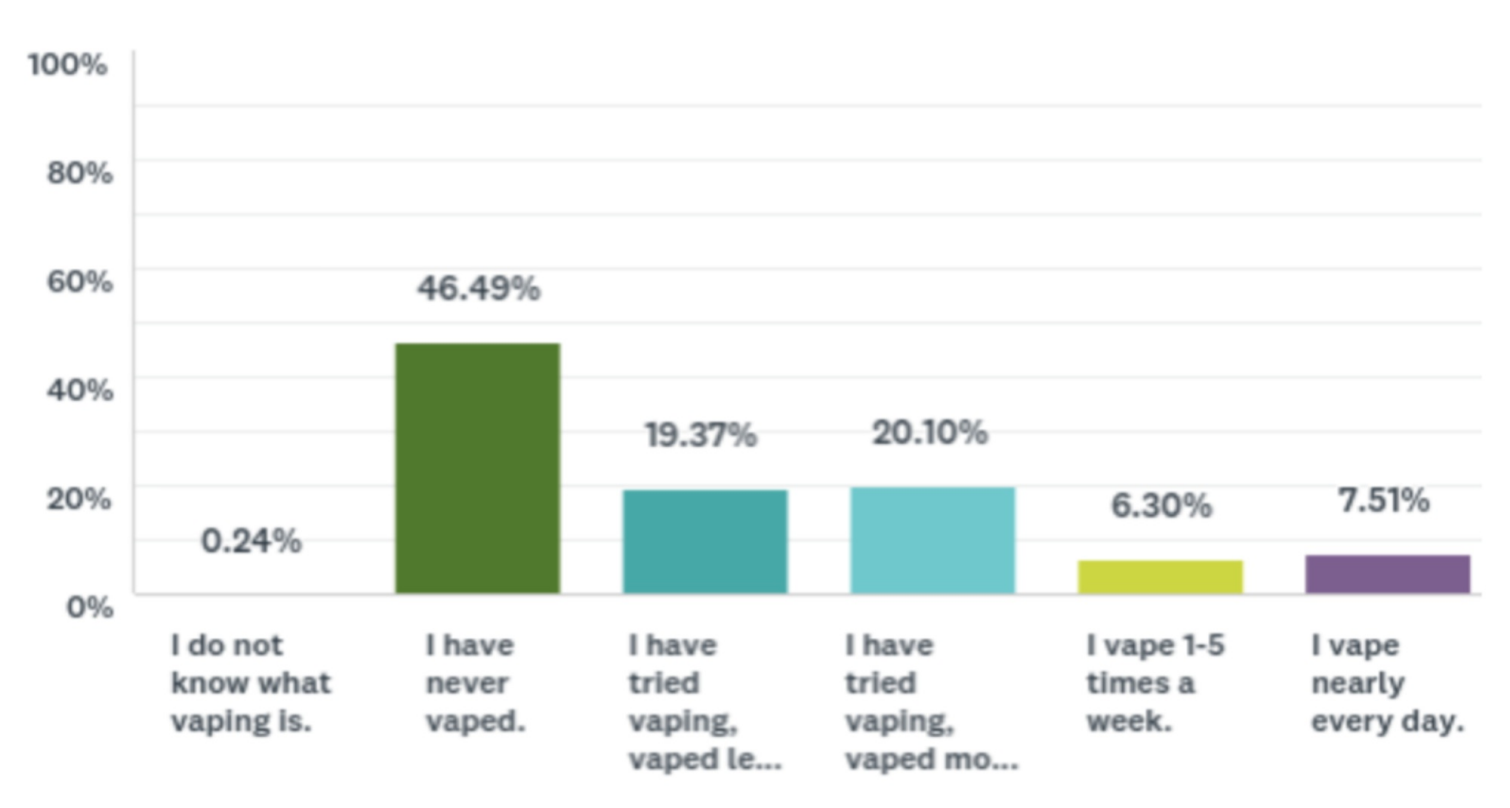
Bar chart demonstrating vaping frequency of the sample Data are shown as percentages.

**Figure 4:**
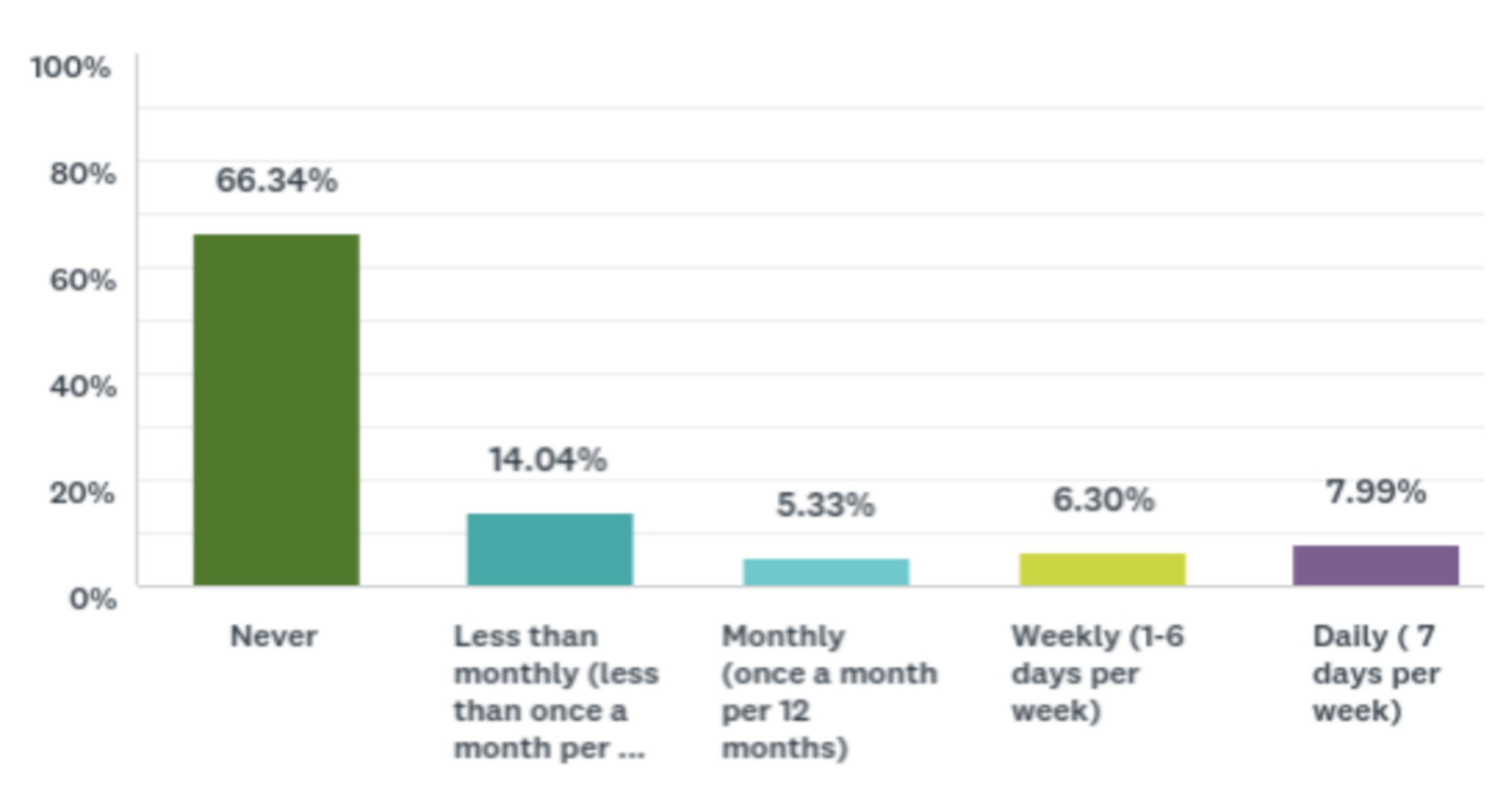
Bar chart demonstrating current vape use among the sample Data are shown as percentages.

**Figure 5:**
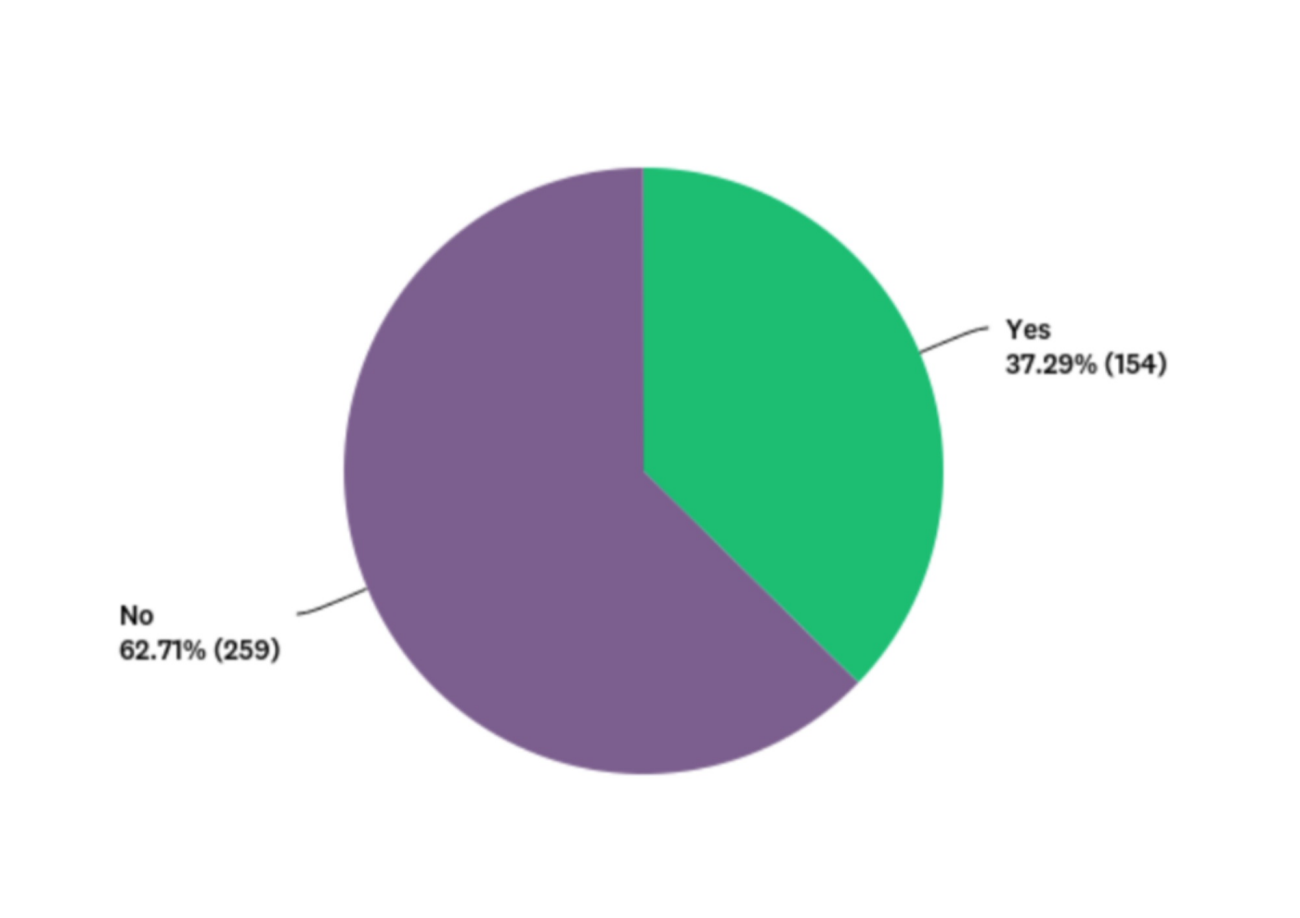
Pie chart showing nominal data regarding whether participants were ever asked about vaping usage by a health care provider Data are shown as percentages.

Less than half the sample (46.49%) had never vaped, and the rest of the participants reported different frequencies of vaping (Figure 3). Data for current vape use among all participants can be found in Figure 4. Data for frequency of drug use among all participants can be found in Table 4. Figure 5 includes data related to whether or not a participant has ever been asked about vaping usage by a health care provider. Data for vaping perceptions and knowledge among all participants can be found in Tables 5 and 6, respectively.

**Table 4:** Reported drug use among participants (N = 413) All data reported as n (%).

Substance	Never	Less than monthly	Monthly	Weekly	Daily
Cocaine	390 (94.43%)	18 (4.36%)	2 (0.48%)	3 (0.73%)	0 (0.00%)
Inhalants	401 (97.33%)	6 (1.46%)	3 (0.73%)	2 (0.49%)	0 (0.00%)
Ecstasy	400 (97.09%)	8 (1.94%)	3 (0.73%)	0 (0.00%)	1 (0.24%)
Hallucinogens	393 (95.39%)	14 (3.40%)	4 (0.97%)	0 (0.00%)	1 (0.24%)
Heroin	409 (99.76%)	0 (0.00%)	0 (0.00%)	0 (0.00%)	1 (0.24%)
Ketamine	410 (99.27%)	2 (0.48%)	0 (0.00%)	0 (0.00%)	1 (0.24%)
Methamphetamines	407 (98.79%)	3 (0.73%)	0 (0.00%)	1 (0.24%)	1 (0.24%)
Marijuana	226 (54.72%)	85 (20.58%)	41 (9.93%)	42 (10.17%)	19 (4.60%)

**Table 5:** Vaping perceptions (N = 413)

Statement	Strongly agree	Agree	Disagree	Strongly disagree
The news has affected my impression of vaping	141 (34.14%)	185 (44.79%)	66 (15.98%)	21 (5.08%)
I have noticed that vaping increases difficulty breathing and coughing	128 (31.76%)	190 (48.15%)	78 (19.35%)	7 (1.74%)
Vaping can reduce stress	39 (9.58%)	191 (46.93%)	120 (29.48%)	57 (14.00%)
Vaping is a health concern	253 (61.86%)	140 (34.23%)	13 (3.18%)	3 (0.73%)
If tobacco was the only flavor offered, people would vape	51 (12.47%)	140 (34.23%)	144 (35.21%)	74 (18.09%)
Drinking alcohol makes someone more inclined to vape	122 (29.83%)	168 (41.08%)	96 (23.47%)	23 (5.62%)
Vaping makes a person more socially acceptable to their friends	34 (8.29%)	88 (21.46%)	188 (45.85%)	100 (24.39%)
If a health care provider advised me to stop vaping, I would quit	181 (44.69%)	152 (37.53%)	63 (15.56%)	9 (2.22%)

**Table 6:** Vaping knowledge (N = 413)

Statement	Strongly agree	Agree	Disagree	Strongly disagree
I can list the ingredients in a vape pod	8 (1.94%)	29 (7.02%)	152 (36.80%)	224 (54.24%)
Vaping can cause lung damage	225 (54.61%)	176 (42.72%)	8 (1.94%)	3 (0.73%)
The ingredients in a vape pod are safe to consume	4 (0.97%)	21 (5.11%)	198 (48.18%)	188 (45.74%)
Vaping can damage a person’s health over time.	231 (56.20%)	162 (39.42%)	12 (2.92%)	6 (1.46%)
Vaping is addictive	236 (57.42%)	164 (39.90%)	7 (1.70%)	4 (0.97%)
Vaping is more harmful than smoking cigarettes	60 (14.63%)	111 (27.07%)	203 (49.51%)	36 (8.78%)

In order to explore relationships between variables, Spearman’s rho correlation coefficient tests were used for all categorical data at an alpha level of 0.05. Table 7 displays all the significant (p < 0.05) Spearman’s rho correlations found in this study.

**Table 7:** Significant Spearman’s rho correlations ^a^p-Value less than 0.05 is significant. ^b^Vaping perception. ^c^Vaping knowledge.

Variables	n	Spearman’s rho	p-Value^a^
Education level x alcohol intake^b^	409	0.111	0.025
Age x news^b^	413	0.175	0.000
Age x reduces stress^b^	407	–0.108	0.030
Age x flavor^ b^	409	0.131	0.008
Gender x breathing/coughing^b^	403	0.139	0.005
Gender x health concern^b^	409	0.183	0.000
Gender x flavor^b^	409	–0.100	0.042
Questioned by provider x news^b^	413	0.109	0.027
Questioned by provider x reduces stress^b^	407	0.136	0.006
Questioned by provider x advised to quit^b^	405	0.114	0.021
Gender x safe to consume^c^	411	–0.114	0.020
Gender x damage health^c^	411	0.138	0.005
Gender x lung damage^c^	412	0.135	0.006
Gender x less harmful cigarettes^c^	409	–0.184	0.000
Questioned by provider x safe to consume^c^	411	0.295	0.000
Questioned by provider x damage health^c^	411	–0.385	0.000
Questioned by provider x lung damage^c^	412	–0.268	0.000
Questioned by provider x addictive^c^	411	–0.169	0.001
Questioned by provider x less harmful cigarettes^c^	409	0.370	0.000
Vape use x news^b^	413	–0.235	0.000
Vape use x breathing/coughing^b^	403	–0.255	0.000
Vape use x reduces stress^b^	407	0.389	0.000
Vape use x health concern^b^	409	–0.408	0.000
Vape use x alcohol intake^b^	409	0.211	0.000
Vape use x advised to quit^b^	405	–0.364	0.000
Vape use x safe to consume^c^	411	0.294	0.000
Vape use x damage to health^c^	411	–0.356	0.000
Vape use x lung damage^c^	412	–0.289	0.000
Vape use x addictive^c^	411	–0.174	0.000
Vape use x less harmful cigarettes^c^	409	0.363	0.000
Vape frequency x news^b^	413	–0.281	0.000
Vape frequency x breathing/coughing^b^	403	–0.269	0.000
Vape frequency x reduces stress^b^	407	0.355	0.000
Vape frequency x Health concern^b^	409	–0.432	0.000
Vape frequency x alcohol intake^b^	409	0.172	0.000
Vape frequency x advised to quit^b^	405	–0.387	0.000
Vape frequency x safe to consume^c^	411	0.268	0.000
Vape frequency x damage to health^c^	411	–0.356	0.000
Vape frequency x lung damage^c^	412	–0.268	0.000
Vape frequency x addictive^c^	411	–0.169	0.001
Vape frequency x less harmful cigarettes^c^	409	0.370	0.000
Vape use x cannabis	413	0.572	0.000
Vape use x cocaine	413	0.245	0.000
Vape use x ecstasy	412	0.143	0.004
Vape use x hallucinogens	412	0.164	0.001
Vape use x inhalants	412	0.140	0.004

## Discussion

This exploratory study helped fill the gap in the literature related to knowledge and perceptions of vaping among young U.S. adults. More significant correlations with perception statements were found than with knowledge statements. This suggests that perceptions of vaping risk play a critical role in the decision to engage in vaping. This finding lends support to our first hypothesis that an association exists between knowledge of the chemicals found in vape pods and vaping. Whether or not such a perception changes as a person ages is unknown. Over 80% of our sample fell between 10 and 34 years of age (Figure 2). Some reasons that incline adults to vape include (1) belief that vaping reduces stress, (2) belief that drinking alcohol makes people more inclined to vape, (3) belief that the ingredients in a vape pod are safe to consume, and (4) belief that smoking cigarettes is more dangerous than vaping. The lack of a significant finding between educational level and knowledge and perceptions of the dangers of vaping suggests that all adults need sound education regarding the dangers of vaping, irrespective of their educational backgrounds. The incidence of EVALI has increased and patients, with this acute condition acutely most often present with severe pulmonary consolidation with respiratory failure [1]. Based on our participants’ responses, we found that if the dangers of vaping were discussed with them by their health care providers, they would be more inclined to quit vaping. This underscores how clinicians can influence vaping behavior changes in patients. Such changes begin with candid conversations about the dangers of vaping between clinicians and patients. Unfortunately, this may be easier said than done. Hurst and Conway [18] conducted a qualitative study on physician attitudes about discussing vaping with patients and documenting vaping usage in the electronic medical record. Many physicians believe that they lack medical knowledge needed to discuss vaping with patients and they rarely screen patients for vaping [18]. In fact, one-third of the physicians in their sample did not hold strong objections to vaping [18]. These data are sobering because they provide reasons why many clinicians avoid vaping conversations with patients.

We found significant positive correlations between vaping and concomitant drug use that support our second hypothesis. There was a moderately strong positive correlation (0.572) between vaping and cannabis use (p = 0.000). Weak positive correlations were found between vaping and cocaine use (0.245; p = 0.000), vaping and ecstasy use (0.143; p = 0.004), and vaping and inhalants (0.140; p = 0.004). Our results align with other studies that have found an association between e-cigarettes and marijuana use in young adults [19]. Researchers found that youth who had used an e-cigarette were 3.5 times more likely to use marijuana compared to youth who had not used an e-cigarette [19]. In a Dutch cross-sectional survey, it was found that access to a variety of flavors is one of the most attractive characteristics prompting initial vape use [12]. In our study, flavor was not found to be a significant factor influencing vape use. This suggests that recent legislation banning the sale of flavored cartridges may not be as effective as intended in deterring vaping [20].

In our cohort, we found that participants who lack knowledge of the content and dangers of vaping are not only more likely to engage in vaping, but they also vape more frequently. This finding supports our third hypothesis that an inverse association exists between knowledge of the dangers of vaping and vape usage. We found a weak positive correlation between vape use and the belief that vaping reduces stress (0.389, p = 0.000). Our data support those of others [14] who also reported that e-cigarette users believe that vaping reduces stress.

This study provides a unique snapshot of the vaping landscape in a cohort of young U.S. adults. Although unknown to us at the time, the data reported in this study were collected during the COVID-19 pandemic [21]. A future study during a non-pandemic time could be conducted and the data compared to ours. We were forced to close the study prematurely as New York City began to shut down. However, our sample size (N = 413) exceeded the minimum identified by our a priori power analysis. A larger sample size could have resulted in more robust results. We do believe that our sample is representative of young, computer-literate U.S. adults. The fact that we were not permitted by the ethical review board to query respondents on their places of residence prevented us from generalizing our results to specific areas both within and outside the United States. Our design was not immune to response bias inherent in survey instruments. Furthermore, we were unable to answer any queries related to unfamiliar terminology on our survey because it was electronically distributed. However, as a result of piloting our survey, we did include operational definitions in simple language to help participants. We believe the internal validity of our study is robust. The homogeneity of our sample may weaken the external validity because the ethics board did not permit us to ask for the geographic locations of participants or their IP addresses. Despite these limitations, our data can provide better direction for future studies on vaping knowledge and perceptions in adults.

## Conclusions

Future studies can be designed to evaluate the efficacy of a vaping cessation “conversation protocol” for clinicians to help them engage in conversations about vaping with patients. Analyzing factors that are most predictive of vaping cessation success would be useful in providing much needed patient education. Future studies can also investigate the associations between vaping and drug use to see which drugs most influence a person’s decision to vape. Whether there exists a synergistic mechanism between the chemicals in vaping products and other drugs that make them more addictive in combination is currently unknown.

Undergraduate medical education should include comprehensive information on the pathophysiology and psychosocial factors of vaping. Such a topic could be included within the neurology, psychiatry, and behavior courses. Such a strategy would expose medical students to the fundamentals of vaping addiction. We also recommend screening for e-cigarettes use during every clinical encounter.

## Appendices

Complete survey instrument (demographic items, vaping knowledge items, and vaping perception items) used in the study protocol.

●      To which gender do you most closely identify with?

○      Male 

○      Female 

○      Transgender female 

○      Transgender male 

○      Gender variant/nonconforming^a^

○      Not listed 

○      Prefer not to answer

^a^Operational definition of gender variant/nonconforming: exhibiting behavioral, cultural, or psychological traits that do not correspond with the traits typically associated with one's sex; having a gender expression that does not conform to gender norms.

●      What is your age range? 

○      18-24 years

○      25-34 years

○      35-44 years

○      45-54 years

○      55-64 years

○      65-74 years

○      75 years or older 

●      Please specify your ethnicity.

○      White/Caucasian

○      African American

○      Asian American

○      Pacific Islander

○      Hispanic/Latino

○      Other

●      What is your highest degree or level of education that you have completed?

○      Some high school, no diploma 

○      High school graduate, diploma or equivalent (example: GED [General Education Diploma]) 

○      Some college credits, no degree

○      Trade school

○      Associate’s degree

○      Bachelor’s degree 

○      Master’s degree

○      Academic doctorate degree (PhD, MD, DO, etc.)

●      Which of the following best describes your current employment status?

○      Employed for wages

○      Self-employed 

○      Out of work and looking for work 

○      Out of work but not currently looking for work 

○      A homemaker 

○      A student 

○      Military 

○      Retired 

○      Unable to work 

●      Your yearly income falls within which range?

○      Less than $25,000

○      $25,000-$50,000

○      $50,000-$100,000

○      $100,000-$200,000

○      More than $200,000

○      Prefer not to say 

●      Are you legally married? 

○      Yes 

○      No 

○      Prefer not to say 

●      How many children do you have?

○      None 

○      1

○      2-4

○      More than 4

○      Prefer not to say 

●      Which languages do you speak fluently? (Check all that apply.) 

○      English 

○      Spanish 

○      Portuguese

○      French 

○      Mandarin

○      Arabic 

○      Other 

○      Prefer not to say 

●      Which of the following statements do you most closely agree with? (Vaping frequency.)

○      I do not know what vaping is.

○      I have never vaped.

○      I have tried vaping, vaped less than 1-5 times in my life, and stopped.

○      I have tried vaping, vaped more than 5 times in my life, and stopped.

○      I vape 1-5 times a week.

○      I vape nearly every day.

●      Which of the following statements do you most closely agree with? (Current vape use.)

○      Never

○      Less than monthly (less than once a month per 12 months)

○      Monthly (once a month per 12 months)

○      Weekly (1-6 days per week)

○      Daily (7 days per week)

●      Which of the following statements do you most closely agree with? Click all that apply.

○      I have used cannabis (marijuana)

○      I have used cocaine

○      I have used ecstasy (MDMA) 

○      I have used hallucinogens

○      I have used heroin

○      I have used inhalants (ex. Poppers, “Huffing”) 

○      I have used ketamine

○      I have used methamphetamines

○      I have never tried using the substances listed above before

○      I have used other substances

●      Have you ever been diagnosed with any of the following medical illnesses?

○      Asthma 

○      Reactive airway disease

○      Chronic bronchitis 

○      Chronic obstructive pulmonary disease (COPD)

○      Recurrent pneumonia 

○      Lung cancer 

○      Other 

○      None of the above 

●      Have you ever been diagnosed with any of the following?

○      Generalized anxiety or panic disorder

○      Major depressive disorder or seasonal depressive disorder

○      Bipolar 1 or Bipolar 2 disorder

○      Substance use disorder 

○      Schizophrenia or schizophreniform or schizoaffective disorder

○      Anorexia nervosa or bulimia 

○      Not listed 

○      Never been diagnosed with a psychiatric medical illness/condition 

●      Have you ever been questioned by a health care provider about vaping?

○      Yes

○      No

Paired knowledge statements:

I can list all the ingredients in a vape pod / I cannot list all the ingredients in a vape pod 

The ingredients in a vape pod are safe to consume / The ingredients in a vape pod are not safe to consume

Vaping can cause lung damage / Vaping cannot cause lung damage

Vaping is addictive / Vaping is not addictive

Vaping is less harmful than smoking cigarettes / Vaping is more harmful than smoking cigarettes

Vaping will negatively affect a person's health over time / Vaping will not negatively affect a person's health over time

Paired perception statements:

The news has affected my impression of vaping / The news has not affected my impression of vaping

Vaping is a health concern / Vaping is not a health concern

Drinking alcohol makes a person more inclined to vape / Drinking alcohol does not make a person more inclined to vape

Vaping makes one more socially acceptable to their friends / Vaping does not make one more socially acceptable to their friends

Vaping can reduce stress / Vaping does not reduce stress

If my doctor or other health care provider advised me to stop vaping, I would quit / If my doctor or other healthcare provider advised me to stop vaping, I would not quit

During any visit to a health care provider in the last 12 months were you questioned about vaping? / During any visit to a healthcare provider in the last 12 months were you NOT questioned about vaping? 

If tobacco was the only flavor offered, people would not vape / People would vape if tobacco was the only flavor offered

I have noticed that vaping increases difficulty breathing and coughing / I have noticed that vaping decreases difficulty breathing and coughing
